# High prevalence of the hotspot complement factor I p.Ile357Met pathogenic variant in Tunisian atypical hemolytic uremic syndrome patients: report of three new cases and review of the literature

**DOI:** 10.3389/fimmu.2025.1623432

**Published:** 2025-08-14

**Authors:** Asma Tajouri, Imen Ayadi, Rimeh BenBrahim, Ikram Mami, Abir Boussetta, Hend Jlajla, Yousr Zerzeri, Haifa Arbi Sassi, Jamila Ben Sassi, Hela Sahli, Lilia Laadhar, Tahar Gargah, Mohamed Karim Zouaghi, Maryam Kallel Sellami

**Affiliations:** ^1^ Department of Immunology, La Rabta Hospital, Tunis, Tunisia; ^2^ LR05SP01 Research Laboratory of Immuno-Rheumatology, La Rabta Hospital, Tunis, Tunisia; ^3^ Faculty of Medicine of Tunis, LR99ES10 Human Genetics Laboratory, University of Tunis El Manar, Tunis, Tunisia; ^4^ Department of Nephrology, La Rabta Hospital, Tunis, Tunisia; ^5^ Department of Pediatrics, Charles Nicolle Hospital, Tunis, Tunisia

**Keywords:** atypical hemolytic uremic syndrome, thrombotic microangiopathy, complement regulatory protein mutation, alternative pathway, factor I

## Abstract

**Introduction:**

Atypical Hemolytic Uremic Syndrome (aHUS) is the prototype of renal diseases secondary to dysregulation of the alternative complement pathway. Our previous studies demonstrated that factor I deficiency appears to be common in Tunisian aHUS patients with the recurrence of a rare variant c.1071T>G (p.Ile357Met) localized within exon 10 of the Complement Factor I (*CFI*) gene. Data in the literature have demonstrated that this variant has a pathogenic effect affecting factor I synthesis and function. The recurrence of this variant in the Tunisian cohort led us to suggest that it could be characteristic of the Tunisian population.

**Methods:**

In this context, we conducted the current study which included 8 adults and three children with suspected aHUS and decreased factor I levels, as well as one relative. We performed molecular investigation by targeting specifically the p.Ile357Met mutation of the *CFI* gene by direct sequencing.

**Results and discussion:**

Interestingly, our results showed that the p.Ile357Met mutation was detected in 3 patients out of 11 (two children and one adult) as well as in one relative. Taking into account the high frequency of this pathogenic variant we could confirm that this latter is a hotspot which could be specific to our population. Thus, it would be interesting to look specifically for this variant in any Tunisian aHUS patient with decreased complement factor I level.

## Introduction

The complement system is an essential component of the innate and adaptive immune system. It plays a major role in the defense against foreign pathogens and the maintenance of homeostasis. The complement system can be activated via three pathways: the classical pathway, the lectin pathway, and the alternative pathway (AP), which converge toward the common terminal pathway, leading to the assembly of the membrane attack complex (MAC). Complement is tightly regulated by soluble and membrane-bound regulators to protect self-tissues from complement-mediated damage (1).

Under normal physiological conditions, the AP in the plasma continuously surveys for pathogen invasion by maintaining a low level of baseline activation through a process called tick-over (2). This process leads to the formation of a fluid phase C3 convertase complex, which is able to interact and cleave native C3 molecules to C3a and C3b (3). As a result, this C3 convertase constantly produces small quantities of C3b, which can attach covalently to nearby surfaces that contain hydroxyl groups. C3b deposition on foreign surfaces such as pathogen membranes leads to its interaction with factor B and factor D, C3 convertase formation and the amplification loop of the AP, and finally pathogen elimination. However, the deposition of C3b on host cells is highly regulated. Host cells express complement regulator molecules on their surface or recruit plasma regulators, thus preventing the formation of C3 convertase. A defect in this regulation results in an excessive activation of the AP responsible for hemolytic uremic syndrome (HUS), which particularly affects the kidney.

HUS is a rare disease included in the group of thrombotic microangiopathy lesions. It is defined as a clinical triad combining mechanical hemolytic anemia, thrombocytopenia, and acute kidney failure (4). Several categories of HUS are described according to the causative agent. Atypical HUS (aHUS) is the least common but most serious form; it affects adults as well as children. It includes complement-mediated aHUS, aHUS due to mutations in the *DGKE* gene, and aHUS of unknown causes (5).

In complement-mediated aHUS, pathogenic genetic variants are identified in approximately 60% of patients and are responsible for a dysregulation of the AP. The list of implicated complement genes includes complement factor H (*CFH*), membrane cofactor protein (*MCP*), and complement factor I (*CFI*), with loss-of-function pathogenic variants in these genes being so far more common than gain-of-function pathogenic variants in complement activators *C3* and *CFB* (6–10). Moreover, anti-CFH autoantibodies have been detected in aHUS-reported cases and are described mostly in children having complete deficiency of factor H-related proteins (CFHR) 1 and 3 secondary to deletion of the *CFHR1* and *CFHR3* genes (11–18). The latter form is considered autoimmune acquired aHUS (19).

CFH, CFI, and MCP regulator factor deficiencies represent 20%–52%, 4%–10%, and 6%–15% of complement-mediated aHUS, respectively. CFI is an 88-kDa multidomain protein synthesized as a single polypeptide chain and four positively charged amino acids (RRKR), which are then cleaved out to yield the heavy chain (50 kDa) and the light chain (38 kDa), which corresponds to the serine protease (SP) domain (20). The SP domain exhibits the classic serine protease catalytic triad: aspartate (Asp), histidine (His), and serine (Ser). These three amino acids form the core of the active site and work together to recognize the substrate (C3b or C4b) and hydrolyze peptide bonds at specific cleavage sites. The function of the CFI mediated by the SP domain is only possible in the presence of a cofactor, which alters the substrate conformation to allow cleavage such as CFH and C4 binding protein (soluble cofactors) or MCP and complement receptor 1 (membrane cofactors).

The CFI protein is encoded by the *CFI* gene located on chromosome 4q25 (21) and encompasses 13 exons (22). To date, more than 500 mutations occurring in the *CFI* gene have been identified (ClinVar, https://www.ncbi.nlm.nih.gov/clinvar) from which more than 50 variants are pathogenic and related to complement-mediated aHUS (ClinVar). Among these variants, 40% are classified as type 1 mutations, which result in a quantitative deficiency and are identified within the SP domain, highlighting the central role that they play in this functional domain (23) [Human Gene Mutation Database (HGMD)].

Our previous data demonstrated that 22% of Tunisian aHUS patients had low plasma CFI levels. Moreover, molecular investigations revealed that three out of the 13 aHUS studied cases (approximately one-quarter of the patients) harbored the already published point mutation c.1071T>G (p.Ile357Met) (rs200881135) localized within exon 10 of the *CFI* gene (24). Data in the literature have demonstrated that p.Ile357Met is a pathogenic variant affecting CFI synthesis and function (25, 26). The recurrence of this variant in the Tunisian population led us to suggest that it could be characteristic of the Tunisian population. In this context, we conducted the current study on 11 patients and one relative referred to the Immunology Department of La Rabta Hospital in the context of a suspicion of aHUS with low plasma levels of CFI. According to our initial findings and in order to facilitate molecular diagnosis and ensure the timely and appropriate management of the patients, we aimed to perform molecular investigation by targeting specifically the p.Ile357Met mutation of the *CFI* gene via direct sequencing.

## Materials and methods

### Patients

Eleven patients (P1–p11) with low CFI were enrolled in this study between 2019 and 2022: eight adults (a code with a capitalized P was assigned to these patients) and three children (a code with a small p was assigned to these cases). Their age ranged from 9 days to 48 years. They were referred to the Immunology Department of La Rabta Hospital for complement exploration in the context of suspicion of aHUS. In addition, in this study, the father (R1) of p7, who was healthy, was included.

### Complement investigations

Complement investigation included the following:

- functional activity of the classical pathway measurement (CH50) according to standard Mayer hemolytic assay (27),
- plasma concentration of C3 and C4 by turbidimetry (The Binding Site, Birmingham, UK), and
- CFH and CFI antigen plasma concentrations by a homemade sensitive double-ligand ELISA method using polyclonal anti-CFI and anti-CFH antibodies (Abcam) (28) for the patients screened before 2021 or by radial immunodiffusion (RID) (The Binding Site, UK) since 2021.

### Molecular studies

#### DNA extraction and CFI sequencing

After informed consent was obtained from the adult cohort and the parents of the pediatric cohort, genomic DNA was isolated from the peripheral blood sample using the standard salting-out extraction procedure (29). Since the exon 10 sequence is short, *CFI* exons 9 and 10 were amplified together by PCR using specific primers, which were chosen to allow the amplification of both exons 9 and 10 but also the 5′ and 3′ splicing sites within the introns flanking these exons. The full details of the primer’s sequences are available upon request. PCR reactions were performed using 0.4 μg of genomic DNA and 5 U of *Taq* DNA polymerase (Promega, Mannheim, Germany) according to the manufacturer’s instructions. PCR cycling conditions were classical with an annealing temperature of 55°C. The PCR products were purified using the innuPREP PCR pure kit (Analytik Jena) according to the manufacturer’s protocol and then sequenced on ABI PRISM 3500 Genetic Analyzer (Applied Biosystems, Foster City, CA, USA). The sequencing was carried out with both sense and antisense primers to confirm the results. *CFI* sequence analysis was performed using the Sequencing Analysis (V6) and SeqScape (V3) software (Applied Biosystems). The reference nucleotide sequence of the *CFI* gene starts from the codon +1 corresponding to the initial Met residue and includes the signal peptide sequence.

## Results

### Genetic study

Direct sequencing of *CFI* exon 9–10 PCR products was performed in 11 patients with suspected aHUS and a relative (R1). Sequencing results allowed us to identify two variants. The first was a homozygous variant NM_000204.5: c.941-49C>G detected in all patients except P8. It consisted of a C-to-G transversion in intron 8 distant of 49 nucleotides from the 5′ end of exon 9.

**Figure 1:**
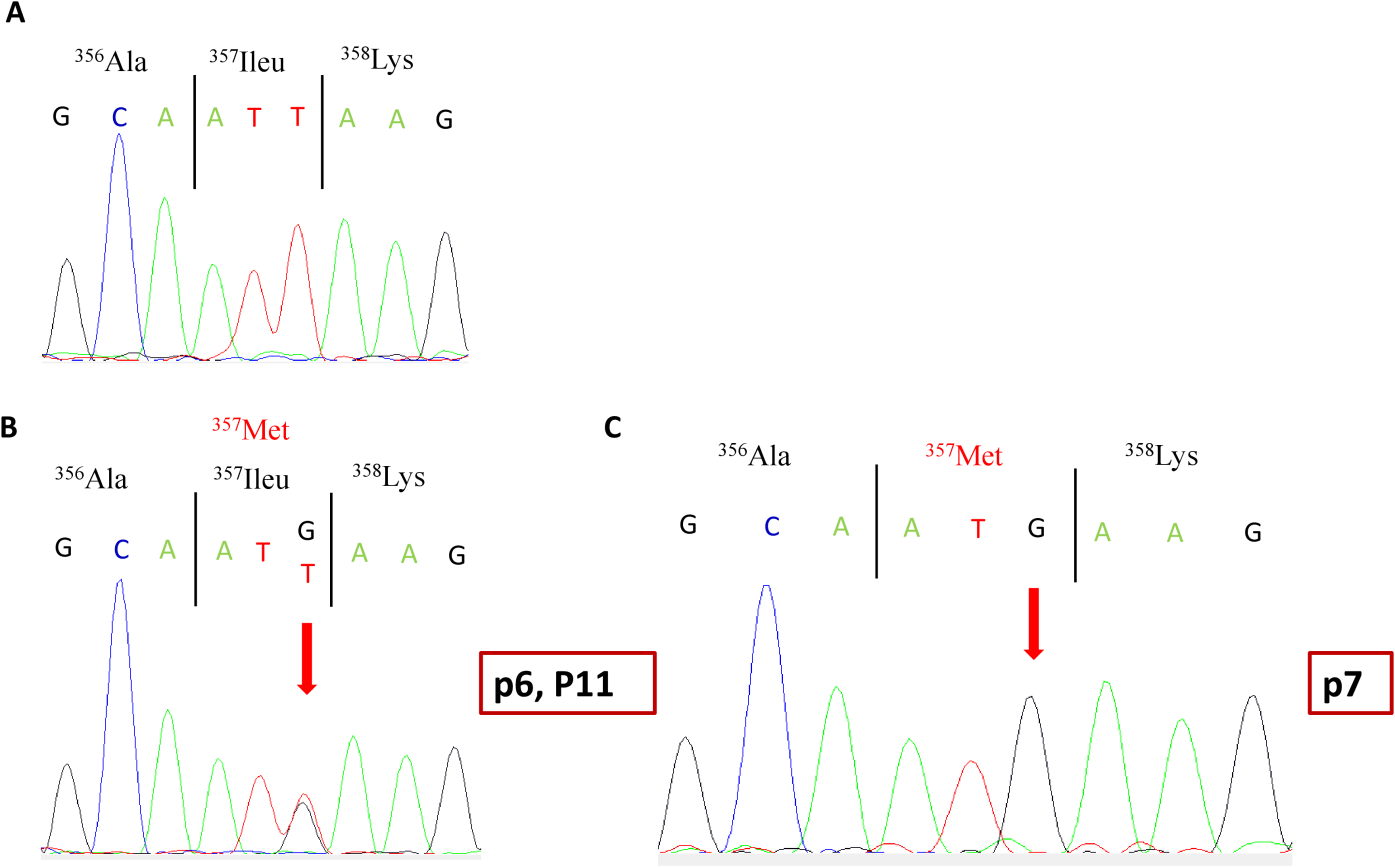
Partial electropherograms of the *CFI* gene exon 10. **(A)** Normal sequence. **(B)** c.1071 T*>*G heterozygous mutation in patients p6 and P11 as well as R1. **(C)** c.1071 T*>*G homozygous mutation in patient p7. The arrow indicates the substituted nucleotide. CFI, complement factor I.

The second variant was a missense variant in exon 10, which consisted of a T-to-G transversion NM_000204.4 (c.1071T>G). It was identified in a homozygous state in p7 and in a heterozygous state in his father R1, as well as in P6 and p11 (**Figure 1**). At the protein level, this variant results in a substitution of a conserved isoleucine (Ile) residue by a methionine (Met) (p.Ileu357Met) in the serine protease domain of CFI. Research in the HGMD (http://www.hgmd.org/) and a review of the literature showed that this variant has already been reported at low allele frequency in the population databases: The Genome Aggregation Database (gnomAD) (https://gnomad.broadinstitute.org) (0.0036%) and 1000 Genomes project (0.0020%). In the Tunisian population, the allelic frequency of this variant in healthy subjects is also very low at 0% (24).

### Clinical characteristics of the patients harboring the c.941-49C>G polymorphism

The clinical data of the patients are summarized in **Table 1**. The biological and genetic data of these patients are summarized in **Table 2**.

**Table 1:** Clinical data of the patients and their relatives.

Patient/sex	Age at diagnosis	Consanguinity	Personal history	Familial history	Triad	Extrarenal involvement	Evolution
P1, M	40 years old	ND	None	Lithiasic nephropathy	Renal failure	MHT	ESRF Proposed for kidney transplantation
P2, F	22 years old	ND	ND	ND	ND	MHT	ESRF Proposed for kidney transplantation
P3, M	24 years old	ND	None	TMA in the cousin FI deficiency	ND	MHT	ND
P4, F	21 years old	ND	Extra-capillary glomerulonephritis	None	Yes, complete	MHT	ESRF
p5, F	1 year old	None	None	None	Renal failure	MHT	ND
P6, M	48 years old	ND	HBP Repetitive angina	Undetermined nephropathy in the mother	Renal failure + thrombocytopenia	MHT	ND
p7, M	4 years old	Yes, 2nd degree	Congenital hip dislocation	None	Renal failure	MHT	ND
P8, M	28 years old	ND	None	None	Yes, complete	MHT	ESRF Proposed for kidney transplantation
P9, M	24 years old	ND	None	Undetermined nephropathy in the maternal grandfather	Renal failure	MHT	ND
P10, M	27 years old	ND	ENT infections	Undetermined nephropathy in the aunt	Renal failure	MHT	ND
p11, F	9 days	ND	None	aHUS in the mother harboring the *CFI* mutation p.Ileu357Met^*^	Thrombocytopenia	None	ND
R1, M**	36 years old	ND	Post-traumatic epilepsy + amygdalectomy	ND	NA	NA	NA

ENT, ear, nose, and throat; ESRF, end-stage renal failure; F, female; M, male; MHT, malignant hypertension; NA, not applicable; ND, not determined; P, adult patient; p, pediatric patient; R, relative; TMA, thrombotic microangiopathy.

* Jlajla et al. (2019) (24).

**R1 is the father of p7.

**Table 2:** Biological and genetic data of the patients and their relatives.

Patient/sex	CH50′	C3″	C4″′	CFI (RIDA or ELISA)	CFH (RIDA or ELISA)	Genetic findings
P1, M	84% (N)	0.844 g/L (N)	0.271 g/L (N)	25.5 mg/L (L)	625 mg/L (H)	*CFI*: c.941-49C>G
P2, F	115% (N)	0.888 g/L (N)	0.33 g/L (N)	21.75 mg/L (L)	449 mg/L (N)	*CFI*: c.941-49C>G
P3, M	133% (H)	0.997 g/L (N)	0.379 g/L (N)	22 mg/L (L)	660 mg/L (H)	*CFI*: c.941-49C>G
P4, F	124% (N)	1.311 g/L (N)	0.274 g/L (N)	14 mg/L (L)	367 mg/L (N)	*CFI*: c.941-49C>G
p5, F	26% (L)	0.618 g/L (L)	0.103 g/L (L)	16 mg/L (L)	330 mg/L (H)	*CFI*: c.941-49C>G
P6, M	104% (N)	1.058 g/L (N)	0.236 g/L (N)	64% (L)	85% (N)	*CFI*: c.941-49C>G *CFI*: heterozygous p.Ileu357Met
p7, M	77% (N)	0.544 g/L (L)	0.203 g/L (N)	24% (L)	113% (N)	*CFI*: c.941-49C>G *CFI*: homozygous p.Ileu357Met
P8, M	131% (N)	0.857 g/L (N)	0.295 g/L (N)	15.25 mg/L (L)	575 mg/L (H)	–
P9, M	92% (N)	0.86 g/L (N)	0.249 g/L (N)	22 mg/L (L)	400 mg/L (N)	*CFI*: c.941-49C>G
P10, M	88% (N)	0.56 g/L (L)	0.304 g/L (N)	21 mg/L (L)	340 mg/L (H)	*CFI*: c.941-49C>G
p11, F	47% (L)	0.636 g/L (L)	0.206 g/L (N)	50% (L)	90% (N)	*CFI*: c.941-49C>G *CFI*: heterozygous p.Ileu357Met
R1, M**	ND	0.908 g/L (N)	0.153 g/L (L)	59% (L)	100% (N)	*CFI*: c.941-49C>G *CFI*: heterozygous p.Ileu357Met

CH50 normal values: 70%–130%. C3 normal values: 0.74–1.62 g/L. C4 normal values: 0.16–0.53. CFH/CFI normal values (ELISA): 85%–130%. CFH normal values in adults (RIDA): 146–553 mg/L. CFH normal values in newborns (RIDA): 178–296 mg/L. CFI normal values in adults (RIDA): 32.3–87.5 mg/L. FI normal values in newborns (RIDA): 15–32 mg/L.

CFI, complement factor I; CFH, complement factor H; ELISA, enzyme-linked immunosorbent assay; F, female; (H), high; (L), low; M, male; (N), normal; ND, not determined; RIDA, radial immunodiffusion assay; P, adult patient; p, pediatric patient.

### Clinical characteristics of the patients harboring the p.Ileu357Met variant

The clinical data of the patients harboring the *CFI* pIleu357Met variant are summarized in **Table 1**. The biological and genetic data of these patients are summarized in **Table 2**.

#### Case report of P6

A 48-year-old man was hospitalized due to a rapid decline in renal function. His medical history revealed a familial background of kidney disease of unknown etiology in his mother. He had no relevant past medical history. The patient's symptoms began 5 months before admission with anorexia, vomiting, weight loss, and a hypertensive surge with a systolic blood pressure of 200 mmHg. Initial laboratory results revealed an elevated creatinine level of 39 mg/L (i.e., clearance of 17 mL/min-MDRD). The patient underwent two emergency hemodialysis sessions due to acute pulmonary edema and a rapid rise in creatinine levels. Laboratory results revealed a hemoglobin level of 9.6 g/dL and a platelet count of 86,000/mm^3^. Peripheral blood smear revealed 1.32% schistocytes. Lactic acid dehydrogenase was elevated at 404 UI/mL. Complement studies revealed a C3 level of 1.058 g/L (normal range: 0.743 to 1.62 g/L), a C4 level of 0.236 g/L (normal range: 0.162 to 0.530 g/L), a CFH level of 85% (normal range: 70%–130%), and a CFI level of 64% (normal range: 70%–130%). Genetic studies revealed the presence of the heterozygous variant p.Ileu357Met in the *CFI* gene. Based on these findings, the diagnosis of complement-mediated aHUS was retained. During hospitalization, type 2 diabetes was discovered due to the presence of diabetic retinopathy on ophthalmological examination. In addition, the patient developed facial cellulitis, which was treated with antibiotics, as well as stage A pancreatitis, which recovered after a symptomatic treatment. Upon discharge from the hospital, the patient remained in terminal renal failure and was placed on antihypertensive treatment, anti-platelet treatment, and two hemodialysis sessions per week.

#### Case report of p7

A 4-year-old boy was hospitalized due to thrombocytopenic purpura. He was born from a second-degree consanguineous marriage, and he had no relevant past medical history. The patient's symptoms began 4 days before admission with asthenia, mucocutaneous pallor, polyarthralgia, and purpuric lesions on the face and thighs in the context of apyrexia. He also presented with hematuria and proteinuria detected by a urine dipstick test. Laboratory results revealed a creatinine level of 48 µmol/L (normal range according to age: 23–37 µmol/L), hemoglobin of 6.3 g/dL, and platelet count of 35,000/mm^3^. Peripheral blood smear revealed 3% schistocytes. Lactic acid dehydrogenase was elevated at 1,800 IU/mL. The direct Coombs test was negative. The myelogram confirmed the peripheral origin of the thrombocytopenia. ADAMTS 13 level was normal, as was the G6PD level, with respective values of 140% (normal range: 50%–150%) and 7.8 U/g Hb (normal range: 4–8 U/g Hb). Complement studies revealed a C3 level of 0.544 g/L (normal range: 0.743 to 1.62 g/L), a C4 level of 0.203 g/L (normal range: 0.162 to 0.530 g/L), a CFH level of 113% (normal range: 70%–130%), and a CFI level of 24% (normal range: 70%–130%). Genetic studies revealed the presence of the homozygous variant p.Ileu357Met in the *CFI* gene. The diagnosis of complement-mediated hemolytic and uremic syndrome was established. The patient initially underwent monthly plasma therapy for 4 years with a good outcome. This therapy was discontinued because of an allergic reaction. Since then, the patient has been treated with eculizumab. In total, he has received six courses of treatment without any particular incident. The patient had a good outcome and recovery of kidney function with the last creatinine level of 36.51 µmol/L.

Notably, R1, the father of p7, was also included in this study. He was a 36-year-old healthy man. Complement investigations revealed a C3 level of 0.908 g/L (normal range: 0.743 to 1.62 g/L), a C4 level of 0.153 g/L (normal range: 0.162 to 0.530 g/L), a CFH level of 100% (normal range: 70%–130%), and a CFI level of 59% (normal range: 70%–130%). Genetic studies revealed the presence of the heterozygous variant p.Ileu357Met in the *CFI* gene.

#### Case report of p11

A 9-day-old newborn girl was investigated for thrombocytopenia without anemia or renal failure. Her medical history revealed a familial background of complement-mediated aHUS in her mother associated with CFI deficiency. The mother had a history of severe pregnancy toxemia causing therapeutic interruption of pregnancy. One month later, she developed post-pregnancy aHUS with the complete triad. She was treated with plasma therapy and immunosuppressive therapy with partial improvement. However, she progressed to renal failure at the hemodialysis stage. The patient died after the third pregnancy. The complement exploration showed a C3 level of 0.745 g/L (normal range: 0.743 to 1.62 g/L), a C4 level of 0.607 g/L (normal range: 0.162 to 0.530 g/L), a CFH level of 65% (normal range: 70%–130%), and a CFI level of 27% (normal range: 70%–130%). Genetic studies revealed the presence of the heterozygous variant p.Ileu357Met in the *CFI* gene. The laboratory results of the newborn girl revealed a hemoglobin level of 15 g/dL and a platelet count of 35,000/mm^3^. Complement studies revealed a C3 level of 0.636 g/L (normal range: 0.743 to 1.62 g/L), a C4 level of 0.206 g/L (normal range: 0.162 to 0.530 g/L), a CFH level of 99% (normal range: 70%–130%), and a CFI level of 50% (normal range: 70%–130%). Performed genetic studies revealed the presence of the same heterozygous variant p.Ileu357Met of the *CFI* gene. We were unable to obtain further data on the clinical presentation, treatment, and clinical progression of the patient, as we could not identify the neonatology department that managed her.

## Discussion

**Figure 2:**
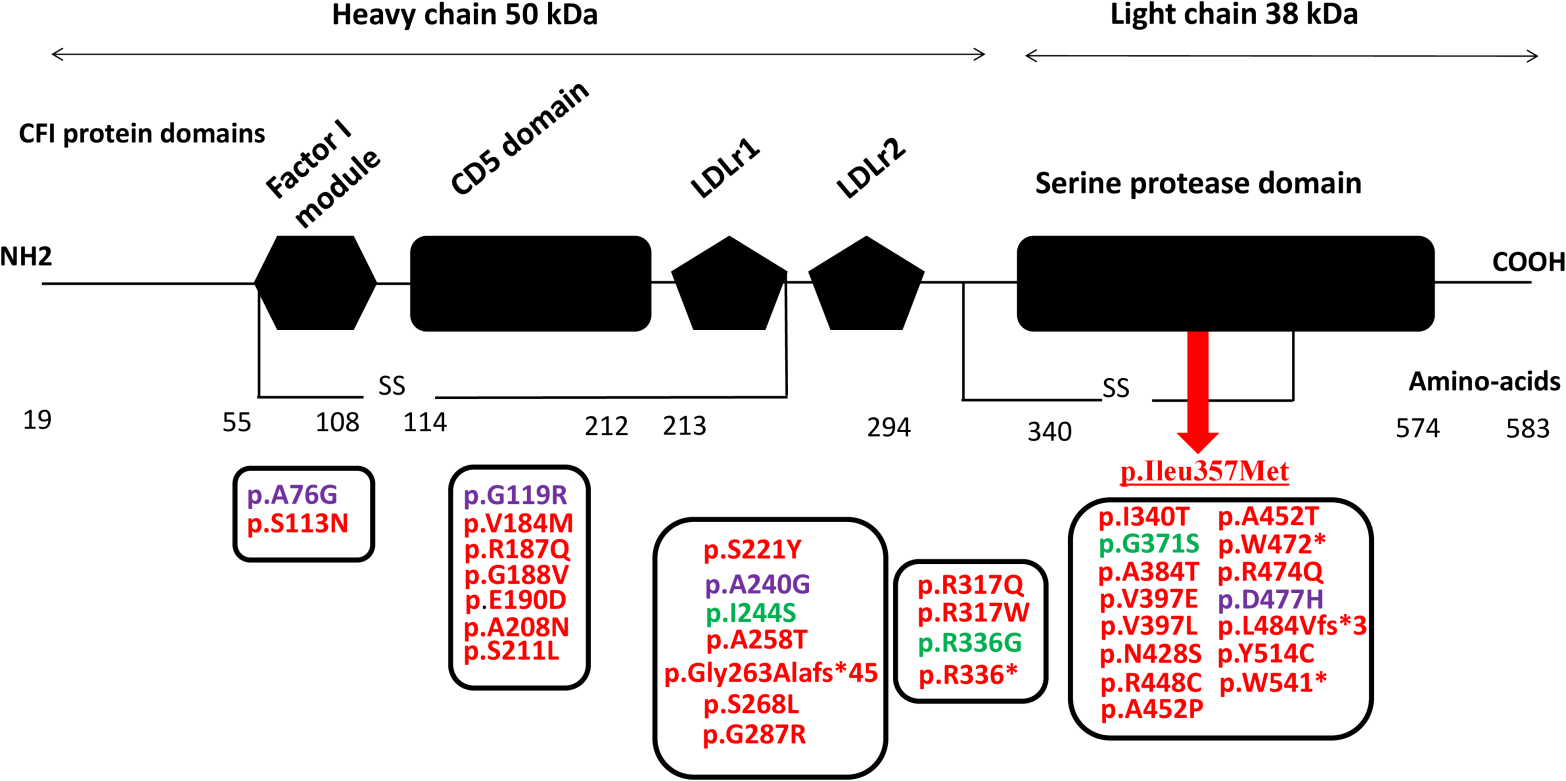
*CFI* pathogenic variants identified in patients with C3G and aHUS (red, aHUS; green, C3G; purple, both C3G and aHUS) localized within the CFI protein domains. The underlined p.Ileu357Met indicates the identified variant within *CFI* gene in the aHUS patients of the current study. CFI, complement factor I; C3G, C3 glomerulopathy; aHUS, atypical hemolytic uremic syndrome.

Since the first description of aHUS cases, a growing number of *CFI* variants have been identified in patients including missense, nonsense, splice-site, and frameshift variants distributed throughout the gene with more than 50 variants being pathogenic and related to complement-mediated aHUS (ClinVar, https://www.ncbi.nlm.nih.gov/clinvar) (**Figure 2**). Notable disease-associated variants include p.Gly119Arg, p.Gly188Val, p.Ser221Tyr, and p.Ile340Thr. These pathogenic variants frequently affect functionally important domains such as the serine protease domain and the scavenger receptor cysteine-rich (SRCR) domain. Functional studies of these variants have demonstrated impaired cofactor-mediated cleavage of C3b and/or C4b, consistent with a loss of regulatory capacity (26, 30–35).

Some variants, such as p.Gly119Arg, are relatively common and have been observed in both familial and sporadic cases. This variant has shown incomplete penetrance, underscoring the importance of environmental triggers (e.g., infections, pregnancy, and transplantation) and potential genetic modifiers in disease expression (35).

Our previous studies demonstrated that CFI deficiency appears to be common in Tunisian aHUS patients with the recurrence of a rare variant c.1071T>G (p.Ile357Met) localized within exon 10 of the *CFI* gene in three out of 11 patients (24). Data in the literature showed that this variant affects CFI synthesis and function (25, 26). The recurrence of this variant in the Tunisian population led us to suggest that this variant could be characteristic of the Tunisian population. In this context, we conducted the current study, which included eight adults and three children with suspected aHUS and decreased CFI levels, as well as one relative. We performed molecular investigation by targeting specifically the p.Ile357Met variant of the *CFI* gene via direct sequencing of exons 9 and 10.

In our study, the genetic analysis allowed us to detect two variants: a polymorphism (c.941-49C>G) and a mutation [NM_000204.4 (c.1071T>G) (p.Ileu357Met)]. The variant c.941-49C>G is a homozygous C>G transversion located in intron 8 distant of 49 nucleotides from the 5′ end of exon 9 of the *CFI* gene. It is a polymorphism that has already been described and classified (by databases) as a benign variation (ClinVar, www.https://www.ncbi.nlm.nih.gov/clinvar/). In the study conducted by Jlajla et al. (2019) (24), this polymorphism was reported in 11 patients and three relatives as well as in 42% of controls (24). Indeed, this polymorphism had been reported in other non-Tunisian cohorts. In fact, Idorn et al published a case report about a daughter and her biological mother, from Europe, who were diagnosed with pregnancy-induced thrombotic microangiopathy and anti-glomerular basement membrane glomerulonephritis, respectively. Both developed end-stage renal disease. The daughter was a heterozygous carrier of the complement factor I G261D mutation, previously described in patients with membranoproliferative glomerulonephritis and atypical hemolytic uremic syndrome. The mother was a non-carrier of this mutation. They shared the same *CFI* homozygous polymorphism c.941-49C>G, which was reported as being benign (36). In our cohort, it was detected in all cases except P8. Given the high frequency of this polymorphism in patients and controls, it is very unlikely to be associated with the disease. The *in silico* prediction software for the effects of intronic variations on splicing [Human Splicing Finder (HSF3)] suggested that this polymorphism has no functional impact (24).

As expected, the second variant was a missense mutation in exon 10, which consisted of a T-to-G transversion NM_000204.4 (c.1071T>G). It was identified in a homozygous state in p7, in a heterozygous state in his father R1, and in P6 and p11. Research in mutation databases and a review of the literature showed that this variant has already been reported. Most variants are usually reported in single individuals; however, the detection of this particular *CFI* variant, p.Ile357Met, is unusually high (9.2% of all *CFI*-associated HUS), which highlights a hotspot area as previously described in *CFH* or *C3* (25). Indeed, Schwotzer et al. confirmed that this variant has been identified as a hotspot mutation in the French HUS registry (25). In essence, we suggest that codon 357 could be a hotspot for mutations due to the nature of the nucleotide sequence of the gene in this region, which has some repeated patterns. This could result in the slippage of the polymerase likely causing the mutation.

In the literature, 16 cases harboring the p.Ile357Met variant associated with homozygous or heterozygous state have been reported, and among them, three underwent kidney transplantation. The clinical and biological characteristics of these patients are summarized in **Table 3**; the LC1–LC16 codes are assigned to these cases for discussion. Thus, our present study brings the total number of aHUS cases harboring p.Ileu357Met to 19.

**Table 3:** Clinical and genetic data of 16 literature cases harboring the CFI mutation p.Ile357Met.

Patient	Origin/race	Age/sex	Personal history	Renal disease	Triad	Extrarenal involvement	Evolution	C3/C4	FI level	Associated genetic abnormalities	Reference
LC1	Tunisia	34 years old/F	None	aHUS in native kidneys	Incomplete	MHT	ESRF	Normal C3 Normal C4	22%	*CFI*: Homozygous p.Ile357Met + 2 intronic polymorphisms (*CFI*): c.71 + 185A>G + c.941-49C>G	(Jlajla et al., 2019) (24)
LC2	Tunisia	37 years old/F	None	aHUS in native kidneys	Complete	Recurrent meningitis Cerebral vasculitis Meningoencephalitis	ESRF	Normal C3 Normal C4	12.5%	*CFI*: Homozygous p.Ile357Met + 2 intronic polymorphisms (*CFI*): c.71 + 185A>G + c.941-49C>G	(Jlajla et al., 2019) (24)
LC3	Tunisia	6 months/F	Repetitive ENT infections	aHUS in native kidneys	Complete	MHT Heart failure	ESRF and death	Low C3 Normal C4	22%	*CFI*: Heterozygous p.Ile357Met *CFH*: Silent heterozygous mutation c.927 A>C + intronic heterozygous mutation c.1520-98G>T + heterozygous polymorphism c.2414–28 C>A + benign heterozygous variation c.3148 A>T	Doctoral thesis of Hend Jlajla « Étude des déficits héréditaires de la voie alterne du complément au cours du syndrome hémolytique et urémique atypique (SHUa) »
LC4	Caucasian	30 years old/M	ND	aHUS in native kidneys	Complete	ND	ESRF	ND	ND	*CFI*: p.Ile357Met *CFH*: R1210C *MCP*: c.286+2T>G	(Bresin et al., 2013) (6)
LC5	Caucasian	6 years old/ M	ND	aHUS in native kidneys	ND	None	ND	Low C3 Normal C4	ND	*CFI*: p.Ile357Met Anti-FH antibody; ΔCFHR1/3	(Westra et al., 2010) (40)
LC6	Croatia	58 years old/ F	Pneumonia 2 times recurrent upper respiratory tract infections	None	NA	None	NA	Low C3 Normal C4	5% (RIDA) 0.4% (ELISA)	*CFI:* Compound heterozygous p.Ile357Met +c.772G>A (exon 5)	(Nilsson et al., 2009) (26)
LC7	ND*	7 years old/ M	SDNS	aHUS in native kidney	Complete	MHT	KFRT KT	Normal C3 Normal C4	43%	*CFI*: p.Ile357Met	(25)
LC8	ND*	28 years old/M	Nephronophthisis	TMA aHUS in native kidneys	Incomplete	None	ESRF Hemodialysis	Normal C3 Normal C4	62%	*CFI*: p.Ile357Met *TTC21B:* not precise variant	
LC9	ND*	19 years old/F	None	aHUS in native kidney	Complete	None	ESRF	Normal C3 Normal C4	94%	*CFI*: p.Ile357Met	
LC10	ND*	39 years old/F	None	aHUS in native kidneys	Complete	None	ESRF Normal KF after 10 months	High C3 and C4	95%	*CFI*: p.Ile357Met	
LC11	ND*	36 years old/F	None	aHUS in native kidney	Incomplete	MHT	KFRT KT C3G in the graft	Normal C3 Normal C4	13%	*CFI*: p.Ile357Met	
LC12	ND*	37 years old/F	None	aHUS in native kidneys	Incomplete	MHT	Hemodialysis KT	Normal C3 Normal C4	64%	*CFI*: p.Ile357Met	
LC13	ND*	32 years old/M	None	aHUS in native kidneys	Incomplete	MHT	Hemodialysis KT	Normal C3 Normal C4	61%	*CFI*: p.Ile357Met	
LC14	ND*	59 years old/F	Unknown kidney disease	*De novo* aHUS after KT	Incomplete	None	KFRT Hemodialysis KT	Normal C3 Normal C4	53%	*CFI*: p.Ile357Met	
LC15	ND*	62 years old/F	Diabetic kidney	*De novo* aHUS after KT	Complete	None	KT	High C3 and C4	73%	*CFI*: p.Ile357Met	
LC16	ND*	59 years old/F	Unknown kidney disease	*De novo* aHUS after KT	Incomplete	None	KFRT KT Hemodialysis	Normal C3 Normal C4	36%	*CFI*: p.Ile357Met	

FI normal values in adults (RIDA): 32.3–87.5 mg/L. FI normal values in newborns (RIDA): 15–32 mg/L.

aHUS, atypical hemolytic uremic syndrome; CFI, complement factor I; CFH, complement Factor H; C3GN, C3 glomerulonephritis; ELISA, enzyme-linked immunosorbent assay; ENT, ear, nose, and throat; ESRF, end-stage renal failure; F, female; KF, kidney function; KFRT, kidney failure requiring replacement therapy; KT, kidney transplantation; LC, literature case; M, male; MCP, membrane cofactor protein, MHT, malignant hypertension; NA, not applicable; ND, not determined; RIDA, radial immunodiffusion assay; SDNS, steroid-dependent nephrotic syndrome; TMA, thrombotic microangiopathy.

*Patients reported in the French cohort characterized by a predominance of the Maghrebi origin.

The 16 cases belong to different ethnic groups and are divided into three Tunisians already reported in our previous study, two Caucasians, one case from Croatia, and 10 cases from a French study. Interestingly, six out of the 10 cases from the French study are Maghrebis. Taken together, the data demonstrate that the p.Ileu357Met variant was reported in 6/24 Tunisian cases: three were reported in the study of Jlajla and her collaborators (24) and three in the present study, as well as six Maghrebis from a French cohort (25). Therefore, comparing the number of Tunisian patients (6) and Maghrebis (6) with the seven cases already reported in the literature of different origins, we can suggest that this variant could be characteristic of Tunisian patients with aHUS.

The age of onset of the disease differs between children and adults. In children, aHUS occurs after the age of 1 year in the case of MCP deficiency, whereas it appears earlier even at birth in children with CFI, CFH, C3, and thrombomodulin (THBD) deficiency (37). The aHUS mediated by the p.Ile357Met variant appeared in adulthood in most cases published in the literature (**Table 3**). However, in the Tunisian cohort, two patients of our study (p7 and p11) and one case from the cohort of Jlajla et al. (LC3) had aHUS during childhood (4 years, 9 days, and 6 months, respectively). Since three patients out of the six Tunisian cases harboring the p.Ile357Met mutation are children, we can assume that this variant can lead to early aHUS regardless of the homozygous or heterozygous variant status.

The penetrance of p.Ileu357Met is incomplete. In fact, patients P6 and p11, carrying the heterozygous mutation, had the disease at different ages. However, relative R1 of p7 is healthy and did not show signs of aHUS despite the fact that he carries the same variant in a heterozygous state. The incomplete penetrance of this variant confirms the polygenic and polyfactorial nature of complement-mediated aHUS. Indeed, the penetrance of aHUS is similar in the groups with *CFH*, *MCP*, and *CFI* mutations and has been estimated to be approximately 50%; i.e., only half of the family members who carry the mutation develop the disease (37). In the study of Bresin et al. (2013) (6), it was shown that the penetrance of aHUS increases significantly and gradually in patients with mutations in one, two, or three genes (6, 38). Trigger events, especially in patients with monogenic variations, can precipitate the onset of disease. The involvement of several genetic abnormalities of the AP regulator genes is demonstrated in three reported cases—LC3, LC4, and LC5—who presented, respectively, a deleterious intronic substitution of the *CFH*, two genetic abnormalities of the *CFH*/*MCP* genes, and an anti-FH associated with *CFHR1* gene deletion (**Table 2**).

A difference in the clinical profile was observed both in patients of our cohort and in those described in the literature carrying the same p.Ile357Met variant. The triad was complete in half of the patients. In our cohort, it was incomplete in the three patients: P6, p7, and p11. Thus, we can assume that the phenotype of aHUS mediated by the p.Ile357Met mutation is heterogeneous.

To evaluate the clinical presentation mediated by the p.Ileu357Met variant as well as prognosis, we relied on the French study of Schwotzer and collaborators since they have reported the largest series including 10 unrelated French cases of aHUS associated with the *CFI* variant p.Ile357Met (25). Among these patients, six cases were Maghrebis. This research group showed that aHUS caused by the *CFI* variant p.Ile357Met is linked to two distinct clinical presentations. The first involves aHUS affecting the native kidneys, characterized by severe renal impairment, frequent malignant hypertension, and a high risk of rapid progression to kidney failure requiring replacement therapy (KFRT), which was also the case of the three Tunisian cases reported by Jlajla et al. (24). However, after kidney transplantation (KT), the primary concern is the development of C3 glomerulopathy (C3G) rather than aHUS recurrence. In fact, C3G is a rare chronic kidney disease mediated by a dysregulation of the AP, which is due to uncontrolled C3 and/or C5 convertase activation, leading to C3 deposits and intra-glomerular inflammation (39). In essence, the authors emphasized the role of the *CFI* variant p.Ile357Met in complement-mediated kidney diseases. Interestingly, like the p.Ileu357Met variant, it was reported that p.Gly119Arg (CD5 domain) and p.Ala240Gly (LDL domain) type 1 variants associated with low CFI plasma levels were also identified in aHUS as well as C3GN patients. In essence, the authors suggested that carrying p.Gly119Arg or p.Ala240Gly variant predisposes for complement-mediated disease depending on the expressivity of the other *CFI* allele and/or the presence of other variants in other genes (32).

The second clinical presentation mediated by the same variant p.Ileu357Met involves *de novo* HUS after KT, triggered by different types and intensities of endothelial cell injury such as infections. In these patients, the clinical profile was considered “*de novo* HUS”, although the initial kidney disease was unknown. Therefore, the authors suggested that the *CFI* variant p.Ile357Met may act as a predisposing genetic factor for various forms of secondary HUS (25).

The therapeutic implications of CFI deficiency triggered by the p.Ileu357Met variant in aHUS warrant careful consideration, particularly with regard to treatment strategies, transplant-related challenges, and the potential for disease recurrence. Plasma therapy, including both plasma exchange and infusion, is a first-line treatment for aHUS; however, its efficiency is highly dependent on the underlying genetic background. In this context, no patient from the French cohort received plasma exchange during the acute phase. In contrast, patient p7 of our cohort responded initially well to this treatment. Complement inhibitors such as eculizumab or ravulizumab, which have shown efficiency in preventing disease progression and recurrence by targeting terminal complement activation, are the most widely used Food and Drug Administration (FDA)-approved treatment for aHUS. Notably, none of the patients reported in the French series were treated with eculizumab during the acute phase. Among the 10 cases reported in the French cohort, seven underwent kidney transplantation, which was frequently associated with post-transplant complications, including three cases of graft recurrence, most often presenting as C3G and requiring the subsequent use of eculizumab. Although two patients showed spontaneous remission (one after 2 days and the other after 10 months), this does not rule out future aHUS episodes, particularly since both had their first manifestation of the disease following pregnancy, a known trigger. In conclusion, transplantation remains a commonly pursued option, but in the context of high-risk variations like p.Ileu357Met, the early initiation and continuous administration of eculizumab appear crucial to prevent post-graft complications. Hence, our data as well as literature review of cases harboring the variant argue in favor of a severe form mediated by the p.Ile357Met variant as well as poor prognosis even after KT.

The impact of the studied variant on CFI plasma level and consequently on the regulation of the AP as well as the development of aHUS was confirmed by *in silico* and *in vitro* functional studies, which suggested a deleterious effect on the structure and function of CFI. *In silico* structural studies involving the modeling of CFI were carried out by Nilsson and his collaborators (26). The generated 3D models showed that the p.Ile357Met mutation is located in a hydrophobic and tightly compacted environment. Thus, the substitution of the Ile by the Met generates steric conflicts with the adjacent amino acids, which causes the disruption of the protein structure and poor folding, resulting in intracellular degradation and low serum levels (26). To confirm the results of the *in silico* structural studies and investigate the functional consequences of p.Ile357Met, Nilsson and his colleagues performed *in vitro* functional studies. The performed Western blotting and ELISA techniques showed that a low level of CFI was detected from the serum or plasma of LC6. Subsequently, in order to check whether the CFI present in small quantities is functional, the researchers performed a C3 and C4 *in vitro* degradation test. These studies showed that no degradation of C4b or C3b was detected *in vitro*. The ratio of CFI concentration between cell lysate and supernatant showed that mutant p.Ile357Met is secreted less efficiently than wild-type CFI (26).

Altogether, the performed *in vitro* functional studies confirmed the pathogenicity of the p.Ile357Met mutation buried in the serine protease domain of CFI, which affects the correct folding of the protein and results in its intracellular degradation and a very small amount of the CFI in the patient serum. The low level of CFI found in both our cohort and those described in the literature harboring this variant is consistent with the results of *in vitro* functional analyses.

Taken together, we can assume that p.Ileu357Met is characterized by clinical heterogeneity and poor prognosis requiring KT with a high risk of relapse on the graft and can lead to early HUS during childhood. Thus, preparation for transplantation and especially the prevention of recurrence on a graft are necessary and require the use of an anti-C5 monoclonal antibody (38), which seems to be effective in patients harboring this variant.

Based on our data from the current study as well as the literature, we can hypothesize that the p.Ile357Met variant appears to be characteristic of the Tunisian cohort, which could reflect a founding effect. In this case, targeting this mutation would be an efficient approach to facilitate molecular diagnosis and ensure the timely and appropriate management of Tunisian aHUS patients. Since our conclusions are limited by the small sample size, these data need to be confirmed in a larger cohort of patients with aHUS who have been thoroughly studied to exclude other forms of HUS. Our immunology department in La Rabta is considered a reference laboratory in complement system investigations. All cases of suspected complement-mediated HUS are referred to our laboratory, which will allow us to have larger cohorts. As prospects, we aim to investigate the age estimation of the p.Ileu357Met variant to give new insight into its origin and retrace the common ancestor.

## Data Availability

The original contributions presented in the study are included in the article/**Supplementary Material**. Further inquiries can be directed to the corresponding author.
