# Supplementary material for: High prevalence of the hotspot complement factor I p.Ile357Met pathogenic variant in Tunisian atypical hemolytic uremic syndrome patients: report of three new cases and review of the literature
Source: Front Immunol. 2025 Aug 14;16:1623432. doi: 10.3389/fimmu.2025.1623432 (PMC12390990; doi:10.3389/fimmu.2025.1623432)
Supplement: Supplementary file 1 [file Table1.docx]

**Supplementary material:**

In addition to aHUS, *CFI* mutations have been implicated in other complement-related disorders, including age-related macular degeneration (AMD) and C3 glomerulopathy, further illustrating the pleiotropic effects of dysregulated complement activity. In this context, p.Arg474Gln variant has been reported in AMD cohorts with normal FI levels (18, 42, 43) as well as in aHUS cohort with low FI levels associated with another type 1 variant p.Ala258Thr (44). Performed *in vitro* functional studies data showed that p.Arg474Gln affects FI cofactor activity with all three cofactors (FH, MCP, sCR1), making p.Arg474Gln a type 2 pathogenic variant. No functional defects were observed with p.Arg187Gln, p.Asp208Asn and p.Ser221Tyr variants associated with normal FI levels.

Zhang et al, 2022 also noticed that two other variants p.Ala76Gly and p.Asp477His, identified in both C3GN and aHUS patients, were associated with much higher FI levels in C3G as compared to aHUS patients. Furthermore, FI functional assays showed normal C3b cleavage in all patients(1). In essence, the authors required performing further research to understand this variability and classified both variants as variants of unknown significance. In essence, functional validation of variants of uncertain significance (VUS) through *in vitro* assays remains critical for establishing pathogenicity. Comprehensive genetic panels, including *CFI* along with other complement genes (e.g., *CFH*, *CD46*, *C3*, *CFB*), have become a standard component of diagnostic evaluation for patients presenting with TMA of unclear etiology

1. Zhang Y, Goodfellow RX, Ghiringhelli Borsa N, Dunlop HC, Presti SA, Meyer NC, et al. Complement Factor I Variants in Complement-Mediated Renal Diseases. Front Immunol. 2022;13:866330.
